# Aberrant Resting-State Cerebellar-Cerebral Functional Connectivity in Unmedicated Patients With Obsessive-Compulsive Disorder

**DOI:** 10.3389/fpsyt.2021.659616

**Published:** 2021-04-23

**Authors:** Keitaro Murayama, Hirofumi Tomiyama, Sae Tsuruta, Aikana Ohono, Mingi Kang, Suguru Hasuzawa, Taro Mizobe, Kenta Kato, Osamu Togao, Akio Hiwatashi, Tomohiro Nakao

**Affiliations:** ^1^Department of Neuropsychiatry, Graduate School of Medical Sciences, Kyushu University, Fukuoka, Japan; ^2^Graduate School of Human-Environment Studies, Kyushu University, Fukuoka, Japan; ^3^Karatsu Red Cross Hospital, Karatsu, Japan; ^4^Department of Clinical Radiology, Graduate School of Medical Sciences, Kyushu University, Fukuoka, Japan

**Keywords:** obsessive-compulsive disorder, cerebellum, functional connectivity, default-mode network, precuneus

## Abstract

**Background:** Although abnormality of cerebellar-cerebral functional connectivity at rest in obsessive-compulsive disorder (OCD) has been hypothesized, only a few studies have investigated the neural mechanism. To verify the findings of previous studies, a large sample of patients with OCD was studied because OCD shows possible heterogeneity.

**Methods:** Forty-seven medication-free patients with OCD and 62 healthy controls (HCs) underwent resting-state functional magnetic imaging scans. Seed-based connectivity was examined to investigate differences in cerebellar-cerebral functional connectivity in OCD patients compared with HCs. Correlations between functional connectivity and the severity of obsessive-compulsive symptoms were analyzed.

**Results:** In OCD, we found significantly increased functional connectivity between the right lobule VI and the left precuneus, which is a component of the default mode network (DMN), compared to HCs. However, there was no correlation between the connectivity of the right lobule VI-left precuneus and obsessive-compulsive severity.

**Conclusions:** These findings suggest that altered functional connectivity between the cerebellum and DMN might cause changes in intrinsic large-scale brain networks related to the traits of OCD.

## Introduction

Obsessive-compulsive disorder (OCD) is characterized by recurrent, intrusive, and distressing thoughts (obsessions) and repetitive behaviors or mental acts (compulsions) that are executed to avoid anxiety or neutralize obsessions. A large number of previous neuroimaging studies have indicated that cortico-striato-thalamo-cortical (CSTC) circuit dysfunction is a pathophysiology of OCD (1, 2).

In recent years, resting-state functional connectivity (rsFC), which is defined as temporal correlations of spontaneous blood oxygen level-dependent (BOLD) signal among spatially distributed brain regions (3) at rest, has been used to analyze neural circuits in the brain. Numerous studies of rsFC have identified intrinsic large-scale brain networks defined as the default mode network (DMN), central executive network (CEN), and salience network (SN). DMN consists of three major subdivisions: the ventromedial prefrontal cortex, posterior cingulate cortex, and precuneus (4). Activities in these cortical regions are decreased during task states (5). CEN is divided into two major subdivisions, the dorsolateral prefrontal cortex and posterior parietal cortex, and activity in them is increased during a wide range of cognitively demanding tasks (6, 7). SN consists of major two regions, the dorsal anterior cingulate cortex and anterior insular cortex (6, 8). SN works on detecting, integrating, and filtering interoceptive, autonomic, and emotional information (6). In addition, SN plays a role in switching between DMN and CEN (8).

In the last decade, several studies using resting-state data demonstrated not only functional dysconnectivity within the CSTC circuit (9–13) but also abnormal functional connectivity within and among the DMN, ECN, and SN (14–18) in OCD. These studies, however, had mainly focused on the pathophysiology in the cerebrum of OCD.

Meanwhile, a large number of studies revealed that the cerebellum is involved in not only motor function but also cognitive function (19–25). In psychiatric disorders such as mood disorder, schizophrenia, and neurodevelopmental disorders, there is abundant evidence of alteration of the cerebellum (26–29). Furthermore, some neuroimaging meta-analysis studies of patients with OCD demonstrated structural and functional abnormalities in the cerebellum. Hu et al. reported greater gray matter volume in the cerebellum in adult OCD (30). Eng et al. also indicated that the gray matter volume in the cerebellum was greater and activation was reduced during a response inhibition task in patients with OCD (31). However, the precise roles of the cerebellum in OCD pathophysiology are still unknown.

Based on rs-fMRI, the subregions in the cerebellum are coupled with specific cortical networks, and rsFC was shown to mediate executive function, the default mode, and sensorimotor function in healthy subjects (32, 33). Especially, recent study revealed that the cerebellum is two times as involved the frontoparietal network as the cerebral cortex (34).

In recent years, several studies have investigated the altered cerebellar-cerebral functional connectivity in OCD. In the first study, Xu et al. compared the cerebellar-cerebral functional connectivity of 27 patients with OCD with that of 21 healthy controls (HCs) (35). They found that OCD patients showed significantly decreased cerebellar-cerebral functional connectivity in executive control and emotion processing networks. They also demonstrated a positive correlation between OCD symptom severity and functional connectivity spanning the right Crus I in the cerebellum and the inferior parietal lobule in the OCD group. Zhang et al. found decreased functional connectivity among the left Crus II, lobule VIII, and striatum and between the right lobule VII and the right striatum and cingulate in 27 medication-free OCD patients (36). Gao et al. investigated spontaneous brain activity by measuring the fractional amplitude of low-frequency fluctuations and resting-state functional connectivity in 64 medication-free OCD patients. They demonstrated that the OCD patients showed significantly increased functional connectivity between the left dorsolateral prefrontal cortex and the left cerebellum (37).

Although these studies reported alterations of cerebellar-cerebral functional connectivity in OCD, further investigation is needed to verify these results of previous studies because there are still few studies of cerebellar-cerebral functional connectivity in OCD.

For this reason, the aim of this study was to verify the alteration of cerebellar-cerebral functional connectivity in a larger number of drug-free OCD patients than previous studies.

## Methods

### Subjects

A total of 109 subjects, including 47 medication-free OCD patients and 62 healthy controls (HCs) matched for age and sex participated in this study. All OCD patients were recruited from the Department of Neuropsychiatry, Kyushu University Hospital, Japan. They were diagnosed primarily using the Structured Clinical Interview for DSM-IV Axis I Disorders-Patient Edition (SCID) and fulfilled DSM-IV criteria. We ensured that none of them met the criteria for any current comorbid Axis I disorder and that all of them also fulfilled DSM-5 criteria for OCD. No OCD participant had taken any psychiatric medication for at least 4 weeks, and nine patients were drug-naïve. HCs were recruited from the local community, and interviewed according to the Structured Clinical Interviewed for DSM-IV non-patient Edition (SCID-NP). None of them had any psychiatric disorder. We excluded participants who had a comorbid axis I diagnosis, neurological disorder, head injury, serious medical condition, or history of drug or alcohol addiction. All of the participants completed an MRI scan, clinical assessment, and neuropsychological test within a few hours on the same day.

This study was approved by the Kyushu University Ethics Committee (No. 27-319). All participants provided written informed consent prior to study commencement.

### Clinical Assessment

To assess the global severity of OCD symptoms, we used the Japanese version of the Yale-Brown Obsessive Compulsive Scale (Y-BOCS) (38). The Hamilton Rating Scales for Anxiety (HAM-A) (39) and Depression (HAM-D, 17-item version) (40) were also used to quantify the degree of anxiety and depression. The Japanese version of the National Adult Reading test (JART) (41) was administered to estimate a participant's verbal intelligence quotient (IQ). We used Student's *t*-test and the chi-square test to compare the demographic and clinical data of the OCD and HCs groups.

### Image Data Acquisition and Preprocessing

The preprocessing and processing of image data acquired in this study were described in our previous study (42). All participants underwent MRI scanning on a 3.0-Tesla MRI scanner (Achieva TX, Phillips Healthcare, Best, The Netherlands) equipped with standard phased array head coils. A T2^*^-weighted gradient-echo echo-planar imaging (EPI) sequence (echo time (TE), 30 ms; repetition time (TR), 2,500 ms; field of view (FOV), 212 × 212 mm; matrix, 64 × 64; slice thickness, 3.2 mm; flip angle, 80°) was acquired from each participant. After an initial 10-s dummy scan, we completed 240 real scans during a 10-min real time scan. During a resting-state fMRI scan, participants were instructed to relax with their eyes opened and watch a presented gray cross. High-resolution T1-weighted anatomical images were also acquired (TE = 3.8 ms; TR = 8.2 ms; FOV 240 × 240 mm; flip angle 8°; slice thickness, 1 mm; inversion time = 1,026 ms) after each EPI image scan. After acquisition of all image data, the arousal level during the scan of all participants was checked by the Stanford-Sleepiness Scale.

We used the CONN toolbox 17.f (http://www.nitrc.org/projects/conn) (43) running on MATLAB R2016b version 9.1.0 (MathWorks, Inc., Natick, MA, USA) on MacOS 10.12.6 to analyze functional connectivity. After discarding the first four volumes, the remaining 236 volumes were preprocessed using the CONN toolbox default spatial and temporal processing. Functional images were slice timing corrections based on the slice order, and realigned and normalized in accordance with the standard Montreal Neurological Institute (MNI) template. Six rigid-body parameters (translational and rotational) were estimated for each subject. The ART scrubbing procedure (https://www.nitrc.org/projects/artifact_detect/) was applied to exclude image artifacts due to head movement using the 97th percentile in a normative sample (with thresholds for motion = 0.9 mm and global signal *z* = 5). We showed invalid scans of each groups applying these thresholds (Table 1). Signal noises from the white matter and cerebrospinal fluid were discerned. Next, fMRI data were band-pass filtered at 0.008–0.09 Hz, and all functional images were smoothed using a Gaussian kernel of 6-mm full width at half-maximum. There was no significant difference between OCD and HC groups in motion parameters (max motion [*t* = 1.45; *p* = 0.149] and mean motion [*t* = 0.90; *p* = 0.368]). From anatomical image of each participants, we created white matter and cerebrospinal fluid masks in the spatial processing steps. Then BOLD signal noise from the white matter and cerebrospinal fluid (CSF) were discerned applying linear regression of white matter and CSF signal as confounding effects (43). To regress out the anatomical component-based noise, CONN toolbox has implementation of the CompCor method (44) for noise reduction along with the efficient rejection of motion and artifactual scans.

**Table 1:** Cerebellar seeds and coordinates grouped by network (35).

**Cerebellar network**	**Cerebellar seed**	**Side**	**MNI (x, y, z)**
Executive network	CrusI_Exec1_	L	−12, −78, −28
	CrusI_Exec1_	R	12, −78, −28
	CrusII _Exec2_	L	−36, −70, −46
	CrusII _Exec2_	R	36, −68, −44
	LobuleVI _Exec3_	L	−36, −52, −34
	LobuleVI _Exec3_	R	36, −52, −34
Default mode network	CrusI_DMN_	L	−32, −76, −34
	CrusI_DMN_	R	34, −80, −36
Affective-limbic network	LobuleVI _Aff_	L	−26, −64, −34
	LobuleVI _Aff_	R	26, −64, −34
	Vermis _Limbic_	L	−4, −80, −34
Motor network	LobuleV_Mot_	L	−20, −50, −24
	LobuleV_Mot_	R	22, −52, −22

We used the spherical seed regions-of-interest (ROI) defined in a previous study (35) (Table 1), referring to the findings of healthy subjects (32, 33). Each ROI was created in each hemisphere as a 6 mm radius sphere.

Following the preprocessing steps, the blood-oxygen-level-dependent (BOLD) signal time series correlation was calculated between each pair of sources for each participant across the resting-state time series, and then a Fisher z transformation was applied. Seed-based connectivity maps were generated from each seed ROI for each participant.

We investigated the difference in functional connectivity from seed ROIs to whole brain voxels between the OCD and HC groups by using a two-sample *t*-test. The significance level was set at the individual voxel *p* < 0.001, and a cluster-size threshold of *p* < 0.05 false discovery rate (FDR) corrected. Then, we conducted a correlation analysis between the abnormal functional connectivity from group-level comparison and the Y-BOCS total score, obsession score, and compulsive score within the OCD group.

## Results

### Demographic and Clinical Characteristics

Table 2 shows the demographic characteristics of the OCD group and HCs. Both groups were well-matched for age, sex, and handedness. The mean total Y-BOCS score in the OCD group was 25.13 (S.D^.^= 5.73). The mean HAM-D-17 and HAM-A scores were significantly higher in the OCD group than in the HCs (*p* < 0.001).

**Table 2:** Demographic and clinical features.

**Variables mean (S.D.)**	**OCD (*n* = 47)**	**HC (*n* = 62)**	**Statistics**			
			*****χ^2^*****	***t***	***df***	***p-*value**
Age, years	33.30 (11.87)	32.61 (11.04)		0.308	107	0.759
Sex, male/female	18/29	22/40	0.091		1	0.763
Handed, right/left	41/6	60/2	3.578		1	0.059
Estimated verbal IQ^a^	^b^104.20 (8.37)	107.45 (9.26)		−1.864	106	0.065
HAM-D-17	5.09 (4.73)	0.26 (0.65)		6.873	47.3	^**^0.000
HAM-A	6.36 (7.47)	0.40 (1.03)		5.371	47.34	^**^0.000
Y-BOCS total score	25.13 (5.73)	0.03		29.712	46.06	^**^0.000
Obsession subscale score	12.68 (3.16)	0.03 (0.19)		27.249	46.00	^**^0.000
Compulsive subscale score	12.45 (3.30)	0.00 (0.00)		25.493	46.20	^**^0.000
Invalid Scan	10.98 (19.41)	7.35 (14.96)		1.09	107	0.278

*HAM-A, Hamilton Anxiety Scale; HAM-D, Hamilton Depression Scale; Y-BOCS, Yale-Brown Obsessive-Compulsive Scale.*

a *Estimated verbal IQ was measured by the Japanese version of the National Adult Reading Test (JART).*

b *One participant did not complete JART.*

** *p < 0.01*.

### Cerebellar-Cerebellum Functional Connectivity in OCD Group Relative to HCs

**Figure 1:**
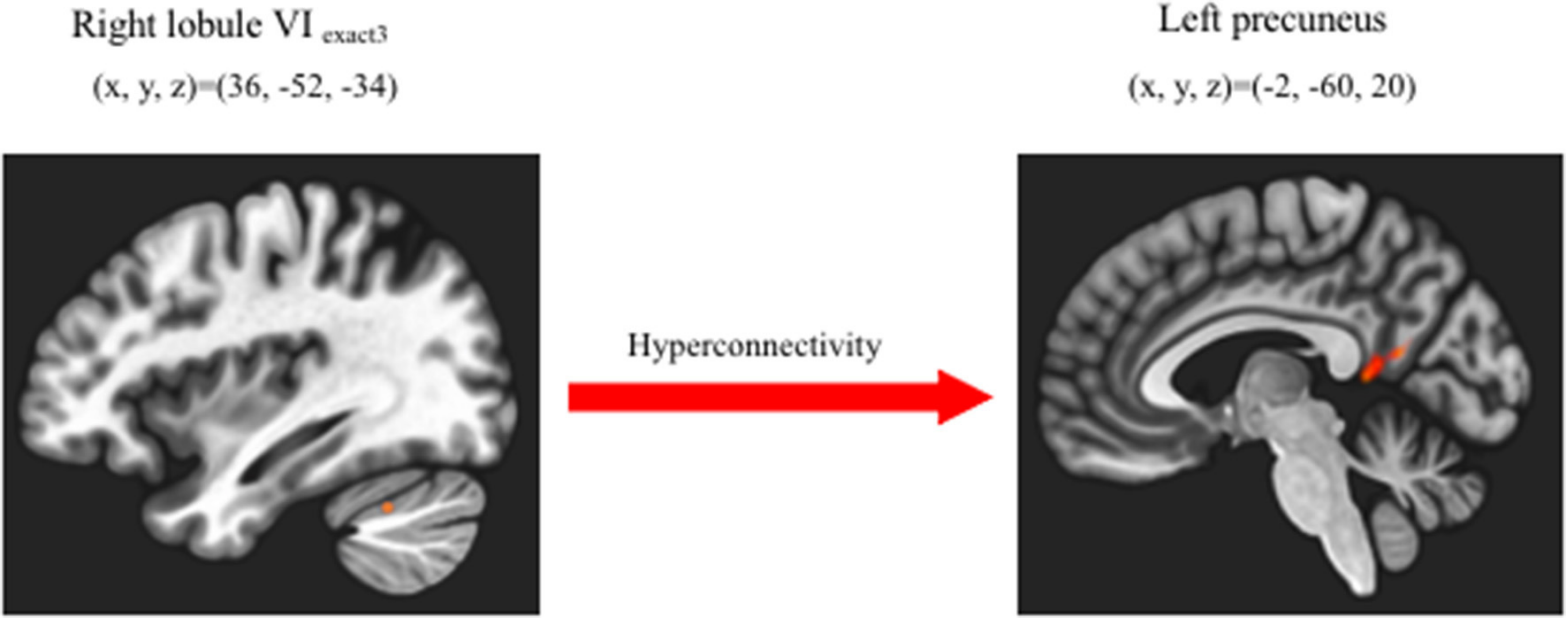
Increased cerebellar-cerebral functional connectivity in OCD group compared with HC group. Patients with OCD showed significantly increased functional connectivity between right lobuleVI_exect3_ and left precuneus than HC (cluster size corrected significance *p* < 0.05 FDR, after applying a per-voxel height threshold of *p* < 0.001).

**Figure 2:**
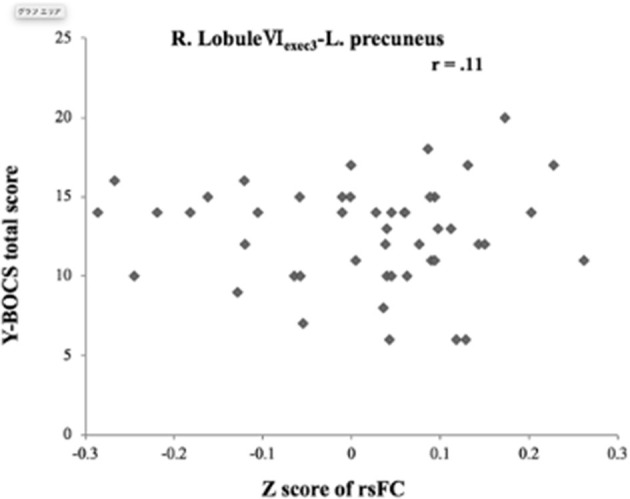
Correlation between altered functional connectivity with severity of obsessive-compulsive symptoms. There was no correlation of increased right lobule VI_exec3_-left precuneus connectivity with the Y-BOCS total score. L, left; R, right; rsFC, resting-state functional connectivity; Y-BOCS, Yale Brown Obsessive-Compulsive Scale.

The OCD group showed significantly increased functional connectivity only between the right lobule VI_exect3_ and the left precuneus [peak MINI coordinate (−2, 60, 20), *p*-FDR:0.005277, cluster size: 195 voxels] (Figure 1). It, however, was not significant difference with Bonferroni correction (*p-*FDR < 0.0038) for adjusting 13 seeds ROIs. No decreased functional connectivity was found in the OCD group compared with HCs. There were no correlations were found between this functional connectivity from the right lobule VI_exact3_ to left precuneus and the Y-BOCS total score (*r* = 0.11) (Figure 2), obsession (*r* = 0.20) score, or compulsion (*r* = −0.014) score within the OCD group (Supplementary Figure 1). For supplemental analysis, within OCD group, we conducted voxel-wise regression analysis from right LobuleVI_exact3_ related to Y-BOCS total scores, while controlling for age and gender (statistical significance was set at a voxel height threshold of *p* < 0.001, and a cluster-size threshold of *p* < 0.05 FDR corrected). Though, there was no brain area that survive statistical significance.

## Discussion

This study showed an increased rsFC between the right lobule VI_exact3_ and left precuneus in OCD patients compared with HCs. There was no correlation between this rsFC and obsessive-compulsive symptom severity. Our findings were different from the results of previous studies that reported hypo- or hyper-connectivity between Crus I and DMN in OCD (35, 45). However, this study had the advantages of a larger number of subjects and more seeds in the cerebellum than previous studies.

Previous studies showed that there were some intrinsic connectivity networks not only DMN, CEN but also visual, somatomotor, attention, limbic networks in the cerebrum (46) and the precuneus participated in paralimbic networks which include subsystems of the DMN (47). We, however, proceed with the discussion based on triple network model hypothesis which was proposed by Menon (48). The precuneus is mapped to the medial parietal cortex and associated with higher-order cognitive processes such as visio-spatial imagery, episodic memory retrieval, and self-processing operations (49). Moreover, the precuneus is one of the brain regions involving the DMN (50–52) which has rsFC with Crus I, Crus II, and Lobule IX in HC (32, 53, 54). Numerous studies revealed alterations of the rsFC within or between the DMN, CEN, and SN in several psychiatric disorders such as schizophrenia, major depressive disorder, and autism (55–60). Menon proposed a triple network model in which a deficit in engagement and disengagement of these core neurocognitive networks play a role in psychiatric disorders (48). In a meta-analysis study of rsFC in OCD, Gürsel et al. demonstrated consistent hypoconnectivity within the DMN, CEN, and SN and general dysconnectivity within the DMN and frontoparietal network, which is involved in CEN, as well as between the frontoparietal lobe, DMN, and SN (60). Therefore, they concluded that the pathological interplay within and between network alterations could underlie core OCD symptoms (60).

**Figure 3:**
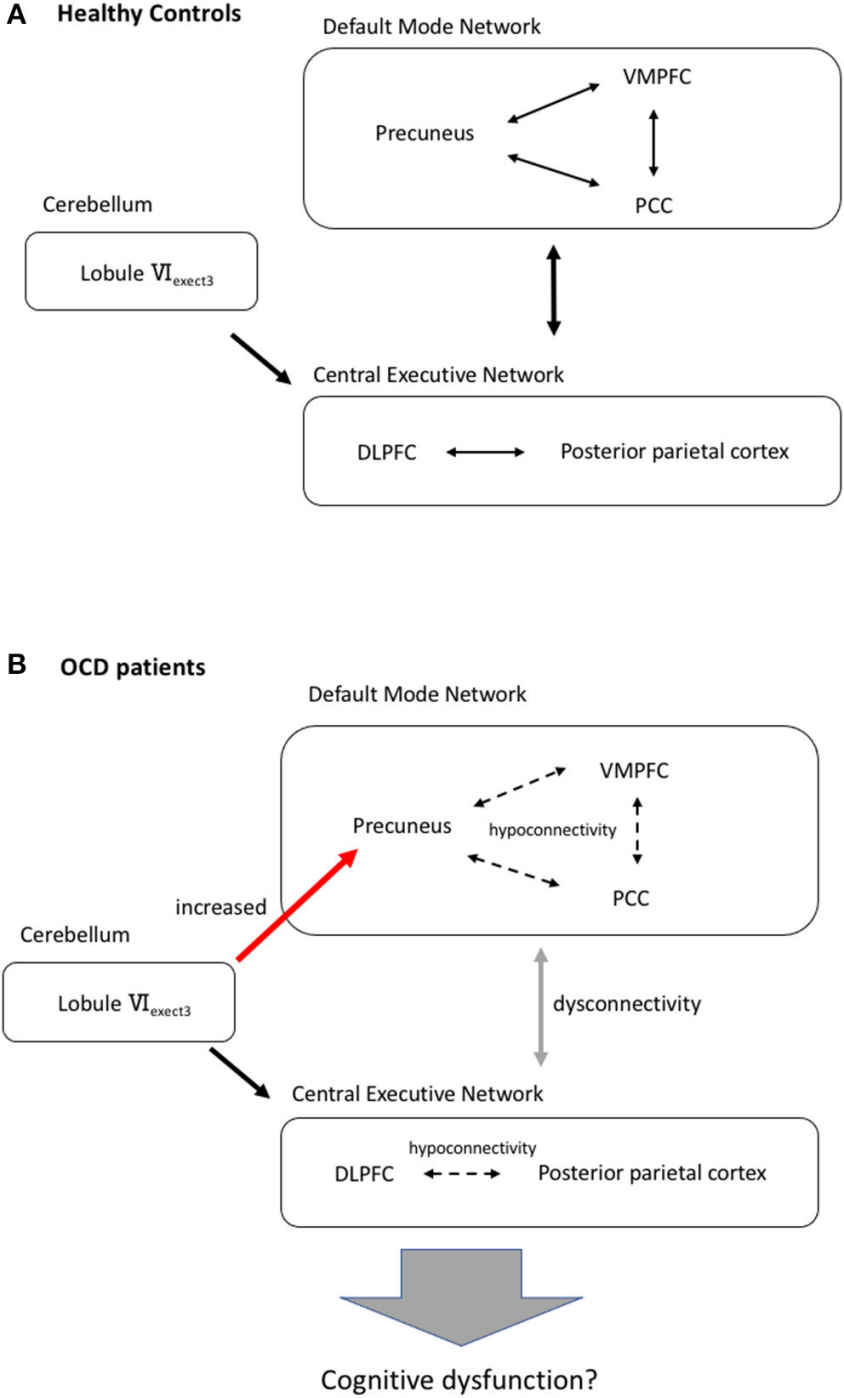
Our hypothesis of aberrant cerebellar-cerebral resting state functional network and cognitive dysfunction of OCD. **(A)** In HC, there is a resting functional connectivity between the lobuleVI_exect3_ and the central executive network. The allow indicates functional connectivity. DLPFC, dorsolateral prefrontal cortex; PCC, posterior cingulate cortex; VMPFC, ventromedial prefrontal cortex. **(B)** Cognitive dysfunction in OCD patients might be associated with increased functional connectivity from lobuleVI_exect3_ to the precuneus, hypoconnectivities in the default mode network and the central executive network and dysconnectivity between these large-scale intrinsic brain networks (60). Dashed line arrow means hypoconnectivity. Red arrow indicates increased connectivity.

Our findings suggest that the aberrant rsFCs might occur not only in the cerebral regions but also in the cerebello-cerebral region in OCD. Patients with OCD have executive dysfunctions, such as working memory, cognitive flexibility, and response inhibition (61, 62). The deactivation of DMN that is associated with these cognitive performances usually occurs when an individual is required to focus attention on an external stimulus in HC (63–65). However, OCD patients have decreased DMN homogeneity (18) in resting conditions and difficulties with deactivation of DMN in non-resting conditions (17). Therefore, we supposed that the increased rsFC between lobule VI, which has resting functional connectivity to the CEN, and the precuneus might relate to interference with the function of DMN and involve the cognitive dysfunction in OCD (Figure 3).

We did not find a correlation between the functional connectivity of the right lobule VI_exact3_–left precuneus and the severity of obsessive-compulsive symptoms measured by Y-BOCS. This result means that the aberrant rsFC between the cerebellum and DMN is not associated directly with the obsessive-compulsive symptoms. DMN relates to response inhibition (66, 67), planning (68), and decision-making (69), which are trait markers for OCD (70). Our results, therefore, might show that this aberrant rsFC is not a state but a trait of OCD patients.

There are several reasons for the differences in the results between the previous study and the current study. First, OCD has heterogeneity (71). It, therefore, has been pointed out that replication of the findings has been variable (71). Second, functional organization of the cerebellum is individual specific (34). Marek et al. (34) revealed that there were differences across individuals from the group average in terms of relative amount of cerebellum associated with each intrinsic cerebral network. Third, there is methodological difference in imaging data analysis between the previous study and current study. We used CONN toolbox (43) which was commonly used in many previous studies, though previous study which was conducted Xu et al. (35) used the Data Processing & Analysis for Brain Imaging (72). We do not think that either of these two methods of analysis is better than the other.

There are several limitations in this study. First, we did not investigate the correlation between aspects of the neuropsychological performance such as response inhibition and aberrant rsFC in the OCD group. Therefore, we could not verify our suggestion that altered cerebellar-cerebral connectivity might relate to the cognitive dysfunction and be a trait of OCD. Second, we did not consider other aspects of OCD heterogeneity, such as the age at onset, duration of the illness, and OCD dimensional symptoms. Future studies with neuropsychological tests and more comprehensive clinical data would validate our study. Third, we had not used the newest validated seed regions which Seitzman et al. (73) had revealed. We, however, use the seed regions which were used in the previous study (35) since the aim of this study was to verify that study. In the future, it is necessary to conduct new analysis using the newest seed regions.

## Conclusion

In conclusion, we found increased functional connectivity between lobule VI and the precuneus at rest in medication-free patients with OCD. There was no correlation between the functional connectivity and severity of obsessive-compulsive symptoms. These findings suggest that aberrant resting state cerebellar-cerebral functional connectivity might be associated with executive dysfunction in OCD patients and be a trait of OCD.

## Data Availability Statement

The original contributions presented in the study are included in the article/Supplementary Material, further inquiries can be directed to the corresponding author/s.

## Ethics Statement

The studies involving human participants were reviewed and approved by Kyushu University Ethics Committee. The patients/participants provided their written informed consent to participate in this study. Written informed consent was obtained from the individual(s) for the publication of any potentially identifiable images or data included in this article.

## Author Contributions

KM designed the study, collected data, and wrote the initial draft of the manuscript. HT collected data, designed the study, and critically reviewed the manuscript. ST, AO, and MK contributed to analysis and interpretation of data. SH, TM, KK, OT, and AH contributed to data collection. TN critically reviewed the manuscript. All authors approved the final version of the manuscript.

## Conflict of Interest

The authors declare that the research was conducted in the absence of any commercial or financial relationships that could be construed as a potential conflict of interest.
