# Supplementary figures and images for: Aberrant Resting-State Cerebellar-Cerebral Functional Connectivity in Unmedicated Patients With Obsessive-Compulsive Disorder
Source: Front Psychiatry. 2021 Apr 23;12:659616. doi: 10.3389/fpsyt.2021.659616 (PMC8102723; doi:10.3389/fpsyt.2021.659616)

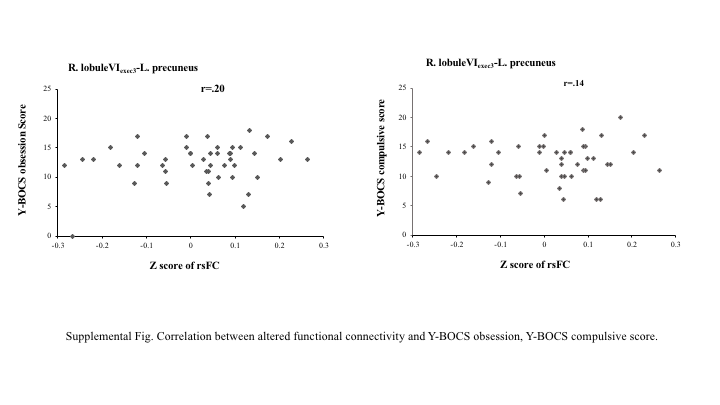

Supplement: Supplementary file 1 [file Image_1.TIFF]
